# Making Mealtime Easier: Nutrition and Texture in Foods for the Elderly with Swallowing Difficulties in Formal and Informal Care

**DOI:** 10.3390/foods15040708

**Published:** 2026-02-14

**Authors:** Cristina M. M. Almeida, Juliana Beltrame, Joana Marto, Lídia Pinheiro

**Affiliations:** 1 Research Institute for Medicines (iMed.ULisboa), Faculty of Pharmacy, University of Lisbon, 1645-003 Lisbon, Portugal; calmeida@ff.ulisboa.pt (C.M.M.A.); jmmarto@ff.ulisboa.pt (J.M.); 2 Faculty of Pharmacy, University of Lisbon, 1645-003 Lisbon, Portugal; julibnascimento@hotmail.com

**Keywords:** dysphagia, elderly, texture-modified diet, rheology, nutrition

## Abstract

Dysphagia, or difficulty swallowing, is a significant issue that impacts 10% to 33% of the elderly population and can lead to serious complications such as aspiration, malnutrition, and weight loss. To overcome these obstacles, there is a critical need for comprehensive rheological data and detailed information on food texture, specifically designed to align with local eating habits and cooking methods. This study aims to develop tables of rheological properties for foods commonly consumed by older adults in Portugal. Additionally, it will assess the impact of water quality on these properties during the cooking process. Based on this data, we will develop texture-modified diets that meet the nutritional needs of elderly dysphagic patients, ensuring they are safe, palatable, and practical for everyday care settings.

## 1. Introduction

Dysphagia, a condition characterized by impaired chewing and swallowing, affects individuals across all age groups and is classified into two types: oropharyngeal and esophageal. Oropharyngeal dysphagia is often associated with neurological disorders or stroke, while esophageal dysphagia results from motility dysfunction. In older adults, dysphagia is frequently linked to presbyphagia, an age-related decline in swallowing function, exacerbated by comorbidities, neurodegenerative diseases, structural changes, medication effects, and muscular disorders such as sarcopenia [1,2].

Swallowing difficulties may also arise from tooth loss, ill-fitting dentures, xerostomia, sensory decline, and reduced orofacial sensitivity, all of which impair mastication and bolus formation. Dysphagia prevalence varies widely depending on the population and assessment methods, ranging from 56% to 66% in nursing homes, 9% to 71% in hospitals, and 5% to 34% in community-dwelling older adults. Managing dysphagia presents multiple challenges, including ensuring safe food textures, nutritional adequacy, palatability, preventing aspiration, providing individualized diets, maintaining hydration, ensuring food safety, promoting psychosocial well-being, and achieving cost-effectiveness. A multidisciplinary approach is essential to address these complex needs and maintain quality of life [3,4].

Malnutrition and dehydration are common in dysphagic patients due to inadequate intake of energy, protein, and fluids. These conditions are associated with immunosuppression, reduced functional capacity, increased infection risk, and higher mortality. Clinical nutrition strategies, such as thickened fluids and texture-modified diets (TMDs), are employed to enhance swallowing safety and efficiency. Thickening agents increase fluid viscosity, thereby reducing the risk of aspiration, whereas TMDs change food consistency through methods such as softening, chopping, or mixing with liquids [5].

TMDs must not only ensure safety but also meet nutritional requirements, including digestibility and bioaccessibility, while maintaining sensory appeal and variety to promote adherence. However, thickened liquids may impair hydration, delay drug absorption, and reduce treatment compliance. Higher viscosity is also associated with increased pharyngeal residue [6].

Water is an inherent component of foods and plays a critical role in determining their safety, stability, quality, and physical behaviour. Its concentration varies widely across food matrices, ranging from less than 1% to over 98%. Fresh and liquid foods typically contain high levels of water, whereas baked or dried products have very low moisture content. In many foods, water is naturally present as part of the intrinsic composition, whereas in others it is intentionally incorporated during processing [7]. In this context, the quality of the water used to prepare TMDs and thickened liquids becomes particularly important. Water composition, including mineral content, pH, and potential contaminants, can influence the effectiveness of thickeners, alter viscosity, and affect the sensory characteristics and safety of the final preparation [8].

Food texture is defined by physical and rheological properties such as hardness, cohesion, viscosity, and elasticity, which influence sensory perception and swallowing mechanics. These properties can be modified through cooking, mechanical processing, the addition of liquid content, or the use of industrial additives such as thickeners and stabilizers. Preferred methods preserve the food’s appearance, color, and flavor to enhance acceptability [5].

Rheological analysis is critical for quality control and safe swallowing, as it evaluates consistency, shear stress, and molecular interactions. However, such analysis typically requires expensive equipment and trained personnel. To overcome these limitations, descriptive classification systems and simplified methods, such as Texture Profile Analysis (TPA), have been developed. TPA simulates the mechanical actions of the teeth and tongue during the first two bites, providing data relevant to oral processing [1,9].

Standardizing food texture classification remains a challenge due to the diversity of foods. Tools like the Dysphagia Outcome and Severity Scale (DOSS) and the Functional Oral Intake Scale (FOIS) help assess dysphagia severity and guide dietary modifications [6].

The TMDs are achieved by modifying the rheological properties of foods. The American Dietetic Association has proposed a four-grade classification of food texture modification, based on the National Dysphagia Diet (NDD). (i) Grade 1: moderate to severe dysphagia (pureed foods); (ii) Grade 2: mild to moderate oral or pharyngeal dysphagia (foods with mechanically modified texture; semi-solids about 0.6 cm); (iii) Grade 3: mild oral and/or pharyngeal dysphagia (soft-solid foods less than 2.5 cm); and (iv) Grade 4: No dysphagia, regular feeding [6]. To extend the oropharyngeal transit time and prevent aspiration, the focus is based on increasing the liquid viscosity to either nectar or honey consistency (51–350 mPa∙s or 350–1750 mPa∙s, respectively) at 25 °C and a shear rate of 50 s^−1^, as established by the NDD [10].

In response to the need for global standardization, the International Dysphagia Diet Standardisation Initiative (IDDSI) was launched in 2012. IDDSI introduced an eight-level framework for classifying texture-modified foods and thickened liquids, using qualitative descriptors and empirical testing. The framework is designed to be universally applicable across ages, cultures, and care settings. Liquids are classified from Level 0 (thin) to thicker consistencies, with mildly thick liquids recommended for mild dysphagia and thicker ones for more severe cases [11,12].

Despite international efforts, countries like Portugal still lack a standardized national classification. The Portuguese Nutrition Association (APN) manual is the most widely used reference, describing soft foods and thickened liquids as nectar, honey, and pudding consistencies [13].

Foods suitable for individuals with dysphagia should be soft, moist, cohesive, and easy to swallow, requiring minimal chewing effort. Ideal TMDs exhibit high viscosity and elasticity, low hardness and stickiness, and leave minimal residue in the oropharynx. Texture can be modified through various methods while maintaining sensory quality to ensure patient acceptance [1].

Due to the high cost and limited availability of texture analyzers, practical alternatives using common utensils, such as forks and spoons, have been adopted. IDDSI test methods, such as the fork drip, spoon tilt, pressure, chopstick, and finger tests, allow for accessible and standardized texture assessment. These tools help reduce subjectivity and support consistent classification of pureed, soft, firm, and solid foods.

Effective dysphagia management requires a comprehensive understanding of food texture, rheology, and nutritional needs. Texture-modified diets, when properly designed and implemented, can significantly improve safety, nutritional status, and quality of life for individuals with swallowing disorders.

Nevertheless, well-designed TMDs (visually/olfactory appealing), when supervised by professionals, support nutritional status in dysphagic patients. Warm meals may aid swallowing and improve acceptance. Standardized terminology enhances safety and communication in multidisciplinary care.

This study aims to characterize the most consumed foods among Portuguese elderly individuals, particularly those with dysphagia, by analyzing their rheological and nutritional properties. It addresses a knowledge gap by providing rheological data for typical preparation methods and examining factors such as the quality of the cooking water.

## 2. Materials and Methods

### 2.1. Selection of Foodstuffs

The food samples were selected based on the food preferences of the Portuguese population, with a particular focus on the dietary needs of the elderly. The selected foods (Table 1) cover all groups of the food wheel used to prepare the two main meals (lunch and dinner): meat and fish, vegetables, pulses, cereals, tubers, and fruit.

The food samples were purchased at supermarkets in Lisbon between January and February 2023. The samples were purchased on the analysis day to avoid prolonged storage periods. All parameters were analyzed in triplicate.

### 2.2. Food Sample Preparation

The food was prepared, cut, and washed according to standard household procedures, including peeling vegetables and tubers, washing them under running tap water and cutting them into uniform-sized slices (Table 2).

Seedless tomatoes, aubergines, and fruits were cut with skin on, dried legumes were soaked for 24 h, and peas were frozen without being soaked. Broccoli and cauliflower were divided into individual flowers, with edible stalks and leaves removed. Flower stems of leafy vegetables were also removed.

### 2.3. Food Cooking

This study focused on water-based food preparation, using demineralized water (DW) and tap water (TW) from Lisbon. Test portions of vegetables (300–500 g), rice (±20 g; rice/water 1:3), meat, and fish were cooked until easily pierced with a fork. Fruit was considered cooked when the skin separated from the fruit. Meat and fish samples were cut into steaks and fillets, with the steaks minced twice and the fish fillets cooked until they were tender and no longer splintered. After cooking, samples were drained, and the volume of water was recorded.

### 2.4. Methods of Food Production and Processing

Food processing involves creating a homogeneous consistency in cooked or fresh foods, such as mashed, grated, crushed, or juiced fruits, which dysphagic patients ingest. For some cooked foods, cooking water was used to facilitate homogenization and viscometer readings, and viscosity analysis was conducted after cooking. Tables S1 and S2 detail different forms of homogenization used.

### 2.5. Sample Analysis

#### 2.5.1. Water

Water quality plays a critical role in food consistency, food viscosity, and overall product performance, as key parameters such as mineral composition, hardness, pH, chlorine content, and suspended impurities directly influence the physicochemical behavior of food systems. At the molecular level, water interacts with major food constituents, such as carbohydrates, proteins, and vitamins, many of which are water-soluble, and contributes to the plasticization of biopolymers such as starch and proteins [8]. These interactions modify hydration, solvation, and structural transitions within the food matrix, thereby affecting processes ranging from gluten network formation in dough to the extraction efficiency underlying coffee’s sensory profile [14]. Variations in water hardness, particularly elevated calcium and magnesium levels, can increase firmness, alter rheology, and extend cooking times in cereal-based products by influencing water diffusion and protein–starch interactions. Chlorine and other dissolved minerals may introduce off-flavors or affect beverage clarity, while particulate impurities contribute to textural heterogeneity. Because these water-driven modifications impact both sensory attributes and processing behavior, maintaining stable and controlled water composition, often through purification or filtration, is essential to ensuring reproducible quality and consistent functional performance in culinary and industrial food applications. Given the importance of water quality, several water parameters were evaluated in both tap and demineralized water, namely, pH, electrical conductivity (EC), turbidity, and total hardness. Cooking water was characterized by its pH and conductivity. Table S3 presents the analytical methods used [15].

#### 2.5.2. Foodstuffs: Fork and Syringe Tests

IDDSI tests confirm product flow or texture in real time for foods and beverages under serving conditions, especially regarding temperature [6,16,17]. This study uses fork and syringe tests for pasty and liquid samples, respectively.

Within the scope of this study and given the types of food used (raw or cooked), only fruit juices belong to the liquid food group.

The fork test requires a fork with long, closely spaced teeth, with a 19 cm-long fork and a 4.5 cm-long tooth.

The sample is crushed using a fork head, with a force exceeding average blood pressure (about 17 kPa, equivalent to the tongue during swallowing) and sufficient to turn the thumbnail white. A syringe flow test was performed using a 10 mL syringe in accordance with the IDDSI standard [17].

#### 2.5.3. Rheological Properties

##### Viscosity Analysis

The viscosity of foods from different food groups was determined using a Brookfield digital rotational viscometer (Ametek Brookfield, Middleboro, MA, USA), model DVE (Ametek Brookfield, Middleboro, MA, USA), with a variable deformation range between 0.3 and 100 revolutions per minute (rpm). The instrument was calibrated daily using polydimethylsiloxane (PDMS) calibration fluid and the Brookfield LV-61 spindle.

After homogenizing hot and cooked samples, with or without the addition of boiling water, they were analyzed using viscometer spindles, and the best spindle/speed combination was selected for the test, yielding satisfactory results between 10 and 100 on the instrument’s percentage torque scale [18].

The process used a Brookfield LV-61 spindle for liquid samples and an LV-64 spindle for pasty samples. The viscosity was measured at 0.3, 2.5, 5, 10, 50, and 100 rpm, and the analyses were performed in triplicate.

The use of a rotational viscometer to analyze samples exhibiting non-Newtonian behaviour constitutes a methodological limitation, which arises not from rotational instruments per se, but from the specific calibration-based rotational viscometer employed, which does not allow control or direct quantification of shear rate and therefore cannot fully characterize the shear-dependent behaviour of non-Newtonian samples [19,20]. As a result, viscosity values should be interpreted with this constraint in mind, acknowledging that a rheometer, or another rotational viscometer capable of controlled-shear measurements, would provide a more complete and representative description of the samples’ rheology.

##### Texture Analysis

The texture profile analysis (TPA) method used a TA.XTplusC texture analyser. The method consisted of a double penetration of a 25.4 mm acrylic cylinder to a depth of 10 mm into the samples packed in cylindrical ceramic containers (38 mm diameter and 42 mm height). The penetration speed was 1 mm/s, and the trigger load was 0.067 N. The Exponent Connect software was used to collect the parameter values. Strength, adhesion, resilience, cohesiveness, gumminess, and chewability were determined. The results presented are the average of the three values [21].

During a TPA test, the instrument records a force–distance curve over two consecutive compression cycles (a simplified representation of the first two bites during the intricate oral processing). Hardness/strength corresponds to the maximum force reached during the first compression. Adhesiveness is obtained from the negative area under the curve during probe withdrawal and reflects the work required to detach the sample from the probe. Resilience is the ratio of the decompression area to the compression area in the first cycle and indicates the instantaneous elastic response. Cohesiveness, calculated as the ratio of the total positive area of the second compression to that of the first, reflects the structural integrity after deformation. Gumminess (hardness × cohesiveness) and chewiness (hardness × cohesiveness × springiness) quantify the energy required to deform and masticate the sample, where springiness represents the elastic recovery. These parameters describe the mechanical behaviour of semisolid matrices and relate directly to their stress–strain responses in double compression. The resulting force–time curves may be further expressed as stress–strain curves, with stress calculated as the measured force divided by the sample’s cross-sectional area, and strain obtained by multiplying the applied strain rate by time [22].

### 2.6. Nutritional Parameters

#### Energy Content

The nutritional and energy value of meals was evaluated based on macronutrients and micronutrients, focusing on lipids, proteins, carbohydrates, fiber, and mineral composition.

The energy content of the meals (kJ/g or kcal/g) was calculated using conversion factors provided in Annex XIV of Regulation (EU) 1169/2011 [23], as outlined in Equation (1).

The P is the protein content (%), CF is the conversion factor, F is the total fat or lipid content (%), C is the experimentally determined total carbohydrate content (%), and DF is the total fibre content (%).


Energy content = (P × CF) + (F × FC) + (C × CF) + (DF × CF)
(1)

The CF for proteins and carbohydrates are 4 kcal/g, for lipids 9 kcal/g, and for fibre 2 kcal/g [16].

The reported values of carbohydrates, protein, lipids, and fibre are deterministic calculations derived from the National Food Composition Table [24] applied to the standard meal composition (soup, main course, and dessert), and not from experimental measurements (Tables S4–S6).

### 2.7. Menu Design

Four menus (I, II, III, and IV) were tailored to meet nutritional requirements for a balanced diet, considering the rheological parameters previously obtained for the target foodstuff. Each menu includes a soup, a main course (served for lunch or dinner), and a fruit option (either fresh or cooked).

Based on the viscosity data, certain meals for dysphagic patients were developed. Menus were designed using the food wheel, taking into account the required daily quantities for their composition and the foods most commonly consumed by Portugal’s elderly population [25].

Four soups (Table S4), main courses (Table S5), and desserts (Table S6) were chosen based on their carbohydrate, fat, protein, and fiber content to ensure optimal nutritional consumption.

Foods with higher and lower viscosities were grouped to ensure balanced textural changes, an organoleptically appealing texture, and acceptability for the dysphagic patient. The olive oil used to prepare meals has a viscosity of approximately 0.04 Pa·s [26].

Therefore, the meals ranged from the least calorific (1502.3 kcal/day) to the most calorific (1716.4 kcal/day), as shown in Table 3.

These recommendations include 100 g for soup and dessert, and 200 g for the main meal. Each of these menus (Table 3) provides the minimal need of 25 kcal/kg body weight/day (1500 kcal/day) for a person weighing 60 kg.

In dysphagic older adults with low physical activity, a pragmatic minimum starting target of ~25 kcal/kg/day can be used to prevent underfeeding, with ~30 kcal/kg/day as the ESPEN guiding value and ~35 kcal/kg/day when malnutrition is present; targets must be individualized by age, sex, physical-activity level (PAL), and disease status [27,28]. This clinical approach complements EFSA population energy requirements for healthy elders with low PAL (≈1800 kcal/day in men; ≈1400 kcal/day in women) [29].

### 2.8. Statistics

Microsoft Excel was used to conduct statistical tests, evaluate rheological and nutritional parameters using basic descriptive statistics and Whisker box plots, and perform one-way analysis of variance (ANOVA) with a significance level of *p* ≤ 0.05, and Pearson’s correlation coefficient to analyze relationships between variables.

## 3. Results and Discussion

### 3.1. Water Analysis

pH can affect the solubilization of several food components, thus increasing or decreasing the viscosity of cooked food (Table S7).

Conductivity is the degree of mineralization of water. The higher its value, the higher the concentration of dissolved salts, which may react with food components to form substances of different consistencies [30]. On the other hand, water electrical conductivity, which reflects the concentration and mobility of dissolved ions, indirectly influences food consistency by modulating key physicochemical processes within the aqueous phase. Higher viscosity reduces ion mobility, thereby lowering conductivity, demonstrating a strong reciprocal relationship between these parameters in liquid foods. This behaviour, reported in sugar solutions, milk systems, and hydrocolloid dispersions, shows that conductivity captures both compositional changes and microstructural constraints that shape rheology. Conductivity also affects biopolymer hydration and network formation by altering ionic strength, thereby influencing protein gelation, starch gelatinization, and hydrocolloid thickening. In concentrated or structured food systems, the ionic composition of water can shift molecular interactions, ultimately modifying viscosity and textural attributes [31].

Turbidity indicates the total content of substances dispersed in the sample, both inorganic and organic [15]. In both water samples, the turbidity value was very low, indicating that this parameter is not a reliable indicator of water quality.

Several ions and organic molecules can increase or decrease the mobility of water molecules in dilute solutions. Small, highly charged cations such as Na^+^, Ca^2+^, Mg^2+^, and Al^3+^ form strongly bound hydration shells in which multiple water molecules become more ordered and less mobile. This local structuring reduces the solution’s overall fluidity relative to pure water. In contrast, larger and more weakly hydrated monovalent ions such as NH_4_^+^, IO_3_^−^, Cl^−^, and NO_3_^−^ interact only weakly with surrounding water and act as structure-breaking (chaotropic) species. These ions disrupt the extent and stability of the hydrogen-bond network between water molecules, thereby increasing water mobility and making the solution more fluid than pure water [32].

The concentrations of calcium and magnesium ions determine water hardness, which does not significantly affect water viscosity; however, it can influence the final appearance, hardness, and viscosity of cooked food [33].

The DW sample is soft, while the Lisbon TW sample is moderately hard, suggesting that food consistency can change during cooking (Table S8). DW and TW change their properties after cooking food. These data may reflect changes during the cooking process, as evidenced by changes in mineralization and the pH of the cooking water for some foods.

As observed in some foods, such as Carolino rice, Arborio rice, carrot puree, pea puree, and Rocha pear, the increase in electrical conductivity of TW used for cooking is followed by an increase in the foods‘ viscosity due to the increase in ions in TW.

Although spinach and chickpea cooking waters showed an increase in EC, they did not exhibit significant changes in viscosity relative to TW, likely due to mineral transfer during cooking [30,34].

During cooking, meat releases soluble substances (nitrogen compounds, minerals, lactic acid, creatinine) from the muscle tissue into the cooking water, which can increase the EC of the cooking water. For pork and chicken steaks, there was an increase in EC and pH, but a decrease in viscosity in TW. This can be explained by the formation of complexes between proteins and calcium and magnesium, which increase water absorption capacity within the myofibril’s structural space [34,35].

### 3.2. Food Viscosity

#### 3.2.1. Selection of Rotational Speed

When setting viscosity criteria for foods intended for dysphagic patients, the choice of shear rate (SR) has long been debated. A shear rate of 50 s^−1^ is the most widely used and accepted reference value in clinical and research settings, as it was adopted by the NDD for defining liquid consistency categories (thin, nectar-like, honey-like, spoon-thick). This standard is widely applied despite the absence of a clear physiological justification for this specific shear rate. The same shear rate has also been recommended by the Japanese Society of Dysphagia Rehabilitation in Japan as the measurement condition for thickened liquids intended for elderly patients with swallowing difficulties, further reinforcing its international use [36,37]. Although viscosity can be easily measured at any constant shear rate using standard rheometers, relying on a single shear rate may be insufficient to describe the behaviour of shear-thinning, thixotropic foods during oral processing, where shear conditions vary dynamically. Several authors recommend performing viscosity measurements over broader shear-rate intervals (e.g., 1–1000 s^−1^), with the selected range depending on the rheometer geometry and the specific objectives of the test [38,39]. Since the relationship between rotational speed (rpm) and shear rate (s^−1^), depends intrinsically on the kinematic conditions imposed during the test and on the specific measurement geometry (e.g., plate–plate, cone–plate, cone angle, concentric cylinders, internal and external cylindrical radii, or the gap between the moving and stationary surfaces), rpm values cannot be generalized across different rheometer types [38,39].

The viscosity and SR values were determined using the experimental setup, the Brookfield viscometer, the spindles, the sample’s rheological behavior, and the equipment’s speed range.

Table S9 lists the selected rotational speeds (rpm) and the corresponding SR values. The SR values were calculated by multiplying the rpm value by the geometry correction factor 0.209 [40]. The foods within each food group are discussed to better understand how water quality affects food viscosity (Table 4 and Table 5).

#### 3.2.2. Cereals and Derivatives, Tubers

As the main component of rice grains, starch accounts for 80% of the total cereal constituents. The different behaviors of the two types of rice are due to the amylose/amylopectin ratio, fat content, and solids concentration [32]. Amylose limits the expansion of starch, while amylopectin is responsible for the gelatinization of starch when heated in an aqueous solution. Viscous solutions are formed as amylose is leached from the granule, increasing the volume of the rice grains while solubilizing the amylose content. After cooling, the amylose molecules undergo recrystallization, while the amylopectin molecules remain unchanged, a consequence of their branched structure. Short-chain amylose increases viscosity, while long-chain amylose and large amylopectin molecules decrease viscosity [41].

The water absorption of Carolino rice was higher than that of Arborio rice [42]. Arborio rice has a chalky or white, opaque core. When chewed but not cooked, Arborio rice’s ability to become rigid explains its higher viscosity. Under the experimental conditions, it was not possible to obtain viscosity values for the higher SR. Due to the presence of calcium and/or magnesium ions (higher hardness), both types of rice displayed increased viscosity in the TW.

The viscoelastic nature of cooked spaghetti may have contributed to the food’s difficult-to-handle, sticky, dense texture. The amount of starch in the spaghetti, which prevents the sample from becoming fluid even when combined with the cooking water, also explains why the cooking water of both samples was more viscous.

Due to its non-Newtonian behavior, the solid content of spaghetti cooking water gradually increases. Pasta’s rheology is influenced by the presence of gluten proteins, which possess cohesive, elastic, and viscous properties that impact its cooking properties. Their structure and molar mass make them physically elastic and fracture-resistant, contributing to pasta’s elasticity [43]. Only spaghetti cooked in TW yielded viscosity data at 2.5 rpm, making comparisons of the results impossible. The best tool for this kind of meal would be a rheometer, as it more accurately captures non-Newtonian behavior, also known as viscoelastic behavior. Therefore, spaghetti can be challenging to swallow because of its viscoelastic behaviour, which can result in choking in people with dysphagia.

Cooked cereals and their derivatives exhibited non-Newtonian shear-thinning behavior due to decreased viscosity, which was related to the starch gel at high concentrations. Potato texture varies with plant cell size and can be influenced by several factors, including pectic compounds and starch content. Internal cell pressures from starch expansion during heating or gelling cause cultivar-specific variations in texture. Starch gelatinization and retrogradation are the primary processes that change the cooked potatoes’ texture [44].

Sweet potatoes have a higher starch content, which may account for the variation in viscosity readings between them and white potatoes cooked in DW. Foods with higher starch concentrations were found to be harder (stiffer) and less tractable after cooking, whereas tubers with lower starch content yielded softer dishes [45]. When heated in excess water at 75–95 °C, starch gelatinizes, increasing cell size. When amylopectin is heated continuously, gelatinization fractures its double helix, making the chains more accessible to enzymatic hydrolysis. Numerous studies have demonstrated that the higher the sugar content and the lower the viscosity, the greater the hydrolysis of starch molecules [46,47].

There were no significant variations in viscosity between purple potato puree cooked in DW and TW. Sweet potatoes contain more total solids than white and purple potatoes, which may improve hardness due to increased structural stability [47]. When white and sweet potato mash were cooked in TW, they became more viscous, which can be attributed to calcium and magnesium ions. Compared to DW, both potatoes had more dry matter and less moisture. Mashed potatoes behaved like non-Newtonian fluids, dropping their viscosity as the cutting speed increased, and might be considered a form of re-fluidization [46].

#### 3.2.3. Vegetables and Fruits

Fruits and vegetables are non-Newtonian foods with a viscoelastic profile [48,49]. Plant cells carrying liquid-phase soluble pectin, which binds to water, solutes, and suspended particles, are dispersed in fruit and vegetable purees. These pectin chains form gels by building a three-dimensional network. Heat treatment can produce both gel formation and pectin degradation (by enzymatic activity, β-elimination, or acid hydrolysis) [50].

Temperature, shear conditions, concentration, particle size distribution, charge, interparticle interactions, soluble pectin and pectins on particle surfaces, hardness and shape, insoluble solids, soluble solids (°Brix), enzymatic activity, polarity, electrolyte concentration, and raw material composition all affect the rheological properties of fruit or vegetable purees [50,51].

The structural changes in horticultural products caused by heating can be explained by the loss of cell turgidity (producing a softer, rubbery texture) and degradation of cell wall components [52].

Pectins with many methoxy groups (—OCH_3_) have been shown to form gels with high soluble solids content. Conversely, pectins with limited methoxy groups form gels in the presence of calcium ions over a broad pH range. The activity of pectin methylesterase at elevated temperatures has been demonstrated to reduce the degree of methoxylation and promote gel formation [50].

The cooked vegetable with the highest viscosity was broccoli, which exhibited a drier appearance and firmer texture than the other vegetables. It is evident that the higher fibre content of turnip results in increased viscosity values.

The viscosity values of buttercup and butternut squash samples cooked in DW at a rotational speed of 2.5 rpm were statistically indistinguishable, with a recorded difference of 12.3 Pa s.

Certain vegetables, including butternut squash, aubergines, broccoli, carrots, and tomatoes, exhibited elevated viscosity levels when cooked in TW, probably due to the presence of calcium and/or magnesium ions in the TW, which enhance the binding of pectin in vegetables due to their higher hardness. For this reason, adding these ions to canned vegetables reduces softening, although this technique is effective only for specific plant tissues [53].

The green leaves (baby watercress, spinach, and turnip greens) had low viscosity values in both types of cooking water (<1.2 Pa s), with the lowest recorded for spinach. This vegetable has a larger leaf area and fewer stalks and veins than baby watercress and turnip greens, which may explain the values obtained.

The acidic pH of tomato puree facilitates the acid hydrolysis of pectin, resulting in a product with lower viscosity. Enzymatic pectin degradation in carrots is limited due to low levels of polygalacturonase [50].

Most vegetables exhibited non-Newtonian shear-thinning behavior, with viscosity decreasing as cutting speed increased [54,55].

Pectin, a constituent of plant cellulose, is subject to degradation through the action of enzymes and chemicals during processing. During heat treatment, pectin solubilisation leads to the softening of the plant tissue, which determines the texture of the resultant puree. Pectin is susceptible to acid hydrolysis due to the acidic pH of the fruit, pH = 3.7 [56].

The fruits cooked in DW showed no significant differences (*p* < 0.05) for the 2.5 rpm speed, with higher values for the Starking apple (62.88 Pa.s) and the Fuji apple (56.16 Pa.s), which also have higher dry matter values. The higher viscosity of the Starking apple can be explained by its slightly higher pH (pH 4.14), which reduces the rate of acid hydrolysis of the pectins.

Fruits with lower viscosity at DW (Gala apple, Golden apple, Rocha pear, Reineta apple) showed an increase in viscosity at TW. Pectic acids, one of the compounds that make up pectin, become insoluble in water when the carboxylate groups combine calcium and/or magnesium, increasing viscosity due to the increase in total soluble solids [53].

Clementine juice (5.28 Pa.s) and orange juice (0.017 Pa.s) had the highest and lowest viscosity values.

No viscosity values were obtained for the Gala, Reineta, Starking, and Rocha pears at 2.5 rpm. The large clusters of fruit cells were not broken up in the crushed fruit, resulting in a nonuniform mixture that could not be read on the viscometer. Under these conditions, the value of the rheological property measured is of limited reliability.

Particle size can be critical in the management of dysphagia because larger particles may cause immediate choking or may become lodged in the cheek or under the tongue, posing a risk of aspiration or subsequent choking. Pureed foods should not be granulated, and particle size should not exceed 1 mm. Crushed fruit is less viscous than grated fruit, and its homogeneity improves feeding conditions for dysphagic patients. Both textures have the characteristics of ready-to-swallow foods, reducing the risk of choking [56,57].

Bananas are commonly consumed as a paste obtained by mashing them with a fork, which has a lower viscosity (0.216 Pa.s) than that of other fruits. This thick puree is suitable for dysphagic patients, reducing the risk of aspiration.

All fruits showed the same rheological, non-Newtonian, and shear-thinning pattern, with the apparent viscosity decreasing with increasing SR, as reported on fruits and juices [51,58].

All fruit juices evaluated in this study (clementine, orange, Fuji apple, gala apple, Golden apple, Reineta apple, and Starking apple) were classified as IDDSI Level 0 (thin), except for pear Rocha juice, which was classified as IDDSI Level 1 (slightly thick).

This result is consistent with the expected behavior of natural fruit juices: most freshly prepared juices, particularly those that have low pulp content, typically meet the criteria for Level 0, flowing freely through the syringe test within the 10 s threshold.

In contrast, pear Rocha juice demonstrated slightly higher resistance to flow, retaining a small volume in the syringe after 10 s, which fits the criteria for IDDSI Level 1. This behavior likely reflects its naturally higher soluble fiber and pectin content, which contributes to a mildly thicker consistency compared to other fruits.

#### 3.2.4. Meat and Fish

Meat composition varies based on species, age, sex, diet, and anatomical area. Myofibrillar proteins, such as actin, myosin, and actomyosin, play a crucial role in water retention, protein binding, gelling, and emulsification. Protein solubility is influenced by pH, ionic strength, temperature, solvents, dehydration, and mechanical processing. Increased pH increases water permeability, reduces structural components, and increases meat tenderness [59].

The higher the protein content, the greater the viscosity. Pork has 36% protein and has the maximum viscosity when cooked in DW (99.6 Pa.s). Beef has the lowest protein level (21%) and viscosity (9.6 Pa.s) when cooked in DW.

Chicken and pork cooked in DW had viscosities of 28.4 Pa.s and 99.6 Pa.s, respectively, at 2.5 rpm. Chicken and pork cooked in DW showed a significant decrease in viscosity, an increase in pH and electrical conductivity of the cooking water, which can be explained by the increase in the ionic strength of the medium and the protein bonds that complex with magnesium and calcium; in the case of actomyosin, this exposes more binding sites and increases the structural space of the myofibril, which promotes water absorption capacity [56].

Beef has the lowest viscosity in DW because its muscle cells and the proteins extracted by grinding have been broken down. Texture properties are influenced by moisture and fat content, which determine water retention and emulsion stability. Turkey and beef exhibited increased viscosity at TW, accompanied by a significant increase in dry matter, suggesting reduced water absorption by the meat [60].

Fish have less connective tissue than meat and more thermally unstable collagen, which melts when cooked. The qualities of its muscular fibers affect the texture of cooked fish. The two varieties of fish cooked in DW had identical viscosity results. The decrease in viscosity of the two kinds in TW could be attributed to an increase in ionic strength between actomyosin muscle filaments or to the formation of calcium-ion complexes. These improvements enhance the fish’s ability to retain water, resulting in more succulent flesh [60].

The meat and fish samples displayed shear-thinning behavior, with the apparent viscosity decreasing as the cutting speed increased.

#### 3.2.5. Pulses

Pea puree has the lowest viscosity of the pulses, which could be attributed to smaller particle size and weaker attractive forces between molecules after heating. White beans, which require the least force to distort, were chosen for testing. When cooked, white beans rise in soluble solids and hence have a higher dry matter content, which may explain the increase in viscosity. The increased viscosity of pea purée and white beans cooked with TW could be attributed to an increase in the medium’s ionic strength. This enhances electrostatic attraction, which is further strengthened by hydrophobic interactions, leading to molecular precipitation and aggregation. Chickpeas absorbed more water during TW cooking, which could explain the decrease in their viscosity. Some research suggests that the vegetable’s reduced viscosity is attributed to its lower lipid content. Pulses exhibited shear-thinning behavior, with the apparent viscosity decreasing as the cutting speed increased, consistent with findings from a pea research study [59,61,62].

**Figure 1:**
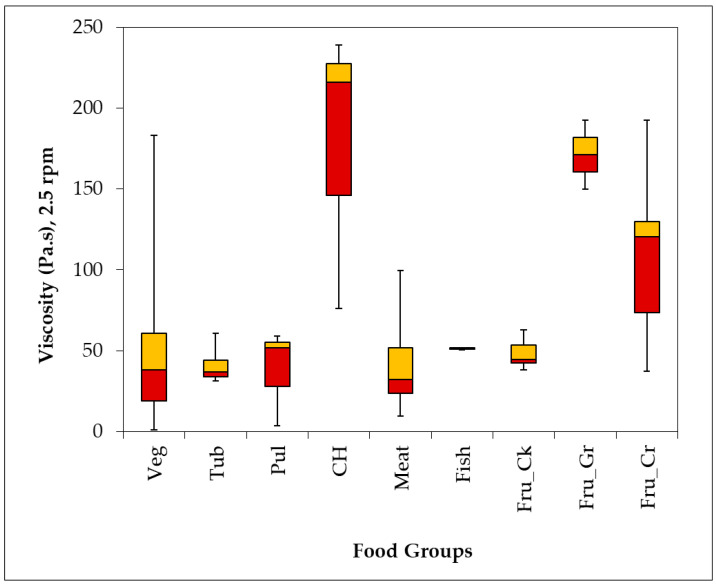
Box-plot of viscosity of the cooked (DW) and fresh foods (grated and crushed) grouped by food groups at 2.5 rpm. Legend: Veg (vegetables), Tub (tubers), Pul (pulses), CH (carbohydrates), Fru_Ck (cooked fruit), Fru_Gr (grated fruit), Fru_Cr (crushed fruit).

Figure 1 shows an overview of the viscosity values obtained for the different food groups at a speed of 2.5 rpm.

Apart from carbohydrates (CH), grated fruit (Fru_Gr) and crushed fruit (Fru_Cr), all other foods had median viscosity values under 50 Pa.s. A high starch content can increase carbohydrate viscosity. In the fruit group, cooked fruit had the lowest viscosity values.

There is no correlation between food viscosity and the corresponding IDDSI scale values (R^2^ = 0.0031). Therefore, the scales should be used independently to compare different foods (Table 1).

### 3.3. Alternative Approaches to Food Consistency

Although highly accurate, rheometry requires specialized instruments and trained personnel, limiting its feasibility for routine use in clinical settings or home care applications. Thereafter, accessible, low-cost methods to assess liquid and semi-solid consistency are essential wherever dysphagia diets are prepared without routine access to rheometers. Empirical consistency, simple gravity-based measurement tools include the Bostwick consistometer, the Ford cup, and the Line Spread Test (LST). The Bostwick consistometer and the Line Spread Test quantify how far a liquid spreads over a set time; a greater spread indicates lower viscosity, while the Ford cup evaluates consistency by measuring how long the sample takes to drain through a standardized orifice [63]. A key drawback of empirical tests is that they cannot fully reflect the dynamic flow behavior of thickened liquids during swallowing. Tools like the Bostwick consistometer provide only a simplified estimate of viscosity, and the Ford cup is strongly influenced by liquid density and fails to capture non-Newtonian characteristics. Nevertheless, evidence from several studies indicates that consistometric testing, particularly with the Bostwick consistometer, emerges as a reliable proxy for clinical thickness targets, provided that its measurement range and sources of variability are properly considered [63,64].

In starch- vs. gum-thickened systems, Xiong et al. demonstrated a robust relationship between IDDSI and consistometric measures, with a linear mapping for starch-based thickeners and a quadratic mapping for xanthan gum-based formulations, reflecting non-Newtonian behaviors and formulation-specific flow mechanisms [64]. This enables conversion of Bostwick distances (cm/30 s) to IDDSI levels within the usable measurement window and supports consistometry as a complementary, quantitative tool alongside the syringe flow test.

Marín Sánchez et al. corroborate this picture from the rheology side: for gum-based thickeners (xanthan, guar, or blends) and a commercial gum containing thickener, Bostwick distance and Line Spread Test show strong (often nonlinear) inverse correlations with apparent viscosity at 50 s^−1^, a shear rate chosen to approximate oropharyngeal conditions. The Ford cup aligns closely with rheometry only at low viscosities (thin/nectar) and becomes inoperable as viscosity increases [63]. Collectively, these data justify the use of consistometers as screening tools that can approximate rheological targets relevant to swallowing.

### 3.4. Texture Profile of Menus

The results of the texture analysis are shown in Table 6. Regarding firmness, except for main course 4, most prepared meals had negative values, suggesting the food was not firm and that the texturometer might be measuring a different firmness parameter.

In a TPA test, the probe compresses the sample (positive force), then withdraws from it. If the sample sticks to the probe during withdrawal, the force curve dips below zero. This results in negative values, which correspond to adhesiveness rather than firmness [21].

Regarding adhesiveness, the negative sign does not indicate an error in the measurement. It reflects the fact that the force recorded during probe withdrawal is in the opposite direction to compression. Larger negative values correspond to samples with greater adhesiveness (i.e., higher stickiness), whereas values that are less negative or close to zero indicate lower adhesiveness [65].

It is common for meat purees to appear coarser, grainier, and with larger particles than vegetable purees, so it may be necessary to add liquid to ensure a moist, cohesive product [66].

Cohesiveness is related to the consistency of the food; if the food does not disintegrate in the first cycle, the value will be close to 1, indicating less fragmentation into particles in the oropharynx and greater safety for dysphagic patients. However, if the food disintegrates completely, the value will be close to zero [67].

Adhesiveness is associated with difficulty swallowing and, in older people, with the sensation of a residue in the mouth. Low adhesiveness values are best for dysphagic patients as they may facilitate swallowing [68].

Elasticity represents one component of a food’s overall viscoelastic behavior and reflects its ability to recover its shape during chewing. Chewability relates to how easily the food can be bitten into. Meat and pulses had high chewability scores, suggesting difficulty biting [67].

Gumminess indicates the work required to turn semi-solid foods into a swallowable cake [69]. Meals containing meat or pulses had high gumminess scores, suggesting difficulty in preparing the food for swallowing.

Negative values of gumminess and chewiness do not represent actual food properties. Such values may arise when the sample is extremely sticky and adheres to the probe, causing it to lift during the test, or when the sample is excessively soft, sticky, or elastic and therefore deforms unpredictably during the compression cycles [21,65].

Physiological factors, such as salivation and oral cavity temperature, which contribute to swallowing, may limit the results obtained with the TPA [68].

Although TPA provides valuable objective information about the mechanical properties of foods, these instrumental results do not fully capture the complexity of oral processing and perception, particularly in elderly individuals with swallowing difficulties [70,71]. Therefore, future studies should incorporate sensory panels to validate the instrumental findings and provide a more comprehensive understanding of product acceptability among older consumers. This combined approach would help ensure that texture-modified meals are not only technically appropriate but also perceived as safe, palatable, and suitable for the target population.

## 4. Conclusions

This work enabled the creation of tables detailing the empirical rheological properties of foods, including viscosity and texture, and incorporated the fork and syringe tests as examples of IDDSI flow tests. The analysed menus provided daily energy values ranging from 1502.3 kcal to 1716.4 kcal, consistent with the standardized portions used in the study (100 g for soup and dessert and 200 g for the main course). Although individual energy needs vary according to age, sex, nutritional status, and physical-activity level, all menus delivered at least ~1500 kcal/day, which corresponds to the clinical minimum intake threshold of ≈25 kcal/kg/day for a 60 kg dysphagic older adult in low-intake settings. This indicates that each menu provides an adequate baseline caloric supply to support nutritional maintenance in individuals with compromised intake, with the understanding that optimal targets should progress toward 30–35 kcal/kg/day when clinically feasible.

If properly balanced and prepared as proposed in this study, the dysphagia diet can replace a regular consistency diet with the same nutritional value, providing a safe, balanced, and pleasant diet for the patient.

Most foods showed differences in viscosity when cooked in different waters (DW or TW), with TW yielding higher consistency. Based on the results and methodology, the rheological behavior of the studied foods can be classified as shear-thinning.

However, carers and health professionals need to utilize these tables to assess their functionality. To complement this study, it would be important to conduct a sensory evaluation of dysphagic patients and, if possible, correlate the rheological parameters with the degree of difficulty in swallowing.

## Figures and Tables

**Table 1:** Foods selected for consistency characterization.

Food Group	Food
Meat and fish	Minced rump steak, chicken steak, turkey steak, pork steak, catfish fillets, Tilapia fillets.
Vegetables	Yellow and butternut pumpkin, baby watercress, leek, aubergine, broccoli (flower), onion, carrot, courgette, cauliflower (flower), spinach, turnip, turnip greens, tomato.
Pulses	Peas, white beans, chickpeas.
Cereals and derivatives, tubers	Rice (Arborio and Carolino), white potatoes, red potatoes, sweet potatoes, spaghetti.
Fruits	Clementines, bananas, oranges, apples (Gala, Golden, Starking, Reineta and Fuji), Rocha pears.
Fats and oils	Olive oil.

**Table 2:** Grouping vegetables by type of cutting.

Portions, 2–3 cm	Slices, 1 cm	Flower	Whole Leaves
Butternut squash Yellow squash White potato Purple potato Onion Apples Turnip Rocha pear Seedless tomatoes	Leek Sweet potato Carrot Courgette	Broccoli Cauliflower	Baby watercress Spinach Turnip greens

**Table 3:** Daily nutritional value corresponding to the two main meals (lunch and dinner).

Daily Menu	Lunch (S + M + D), Kcal			Dinner (S + M + D), Kcal			Menu Kcal/Day
I	S2	M4	D2	S1	M2	D1	1502.3
	39.6	593	64.4	46.4	654.4	104.5	
II	S1	M1	D1	S2	M2	D2	1579.5
	46.4	670.2	104.5	39.6	654.4	64.4	
III	S3	M3	D3	S4	M4	D4	1639.2
	54.3	661.4	104.8	49.9	593	175.8	
IV	S4	M1	D4	S3	M3	D3	1716.4
	49.9	670.2	175.8	54.3	661.4	104.8	

S—soup (100 g); M—main course (200 g); D—dessert (100 g).

**Table 4:** Comparison between the apparent viscosity of the cooked foods in demineralized (DW) and the scale established based on the IDDSI values (*n* = 3).

IDDSI Levels		Food	Preparation	DW		
				*η* (Pa·s) 2.5 rpm	*η* (Pa·s) 5 rpm	*η* (Pa·s) 10 rpm
Level 4	4 A	Butternut squash	Mashed	68.9 ± 3.60	46.2 ± 7.69	24.6 ± 2.51
		Yellow squash	Mashed	56.6 ± 10.42	32.5 ± 12.22	15.8 ± 4.13
		Fuji apple	Mashed	56.2 ± 6.80	31.7 ± 4.61	18.8 ± 1.27
		Gala apple	Mashed	43.4 ± 7.59	24.0 ± 4.97	14.8 ± 2.68
		Golden apple	Mashed	42.0 ± 2.13	26.8 ± 3.90	14.3 ± 0.12
		Reineta apple	Mashed	37.9 ± 4.76	25.1 ± 1.34	15.0 ± 0.81
		Starking apple	Mashed	62.9 ± 3.85	33.2 ± 4.64	20.5 ± 1.26
		Rocha pear	Mashed	45.5 ± 6.28	24.1 ± 4.89	14.1 ± 1.91
	4 B	White potato	Mashed	31.4 ± 16.94	15.0 ± 1.97	8.70 ± 2.57
		Sweet potato	Mashed	38.6 ± 2.69	23.9 ± 2.07	14.6 ± 1.31
		Purple potato	Mashed	34.7 ± 0.17	23.6 ± 6.64	14.2 ± 2.40
		Turnip	Mashed	174.5± 37.51	112.6 ± 3.68	—
	4 C	Carrot	Crushed	60.6 ± 0.51	37.8 ± 9.79	26.3 ± 2.33
		Cauliflower	Crushed	3.09 ± 0.20	1.59 ± 0.39	1.11 ± 0.18
		Courgette	Crushed	38.1 ± 12.70	12.1 ± 3.34	8.17 ± 3.07
		Tomato	Crushed	19.1 ± 1.75	10.1 ± 1.92	6.54 ± 1.52
	4 D	Catfish	Crushed	50.6 ± 25.04	26.3 ± 3.68	14.2 ± 1.23
		Tilapia fish	Crushed	51.7 ± 15.8	21.5 ± 4.04	13.2 ± 1.83
Level 5	5 A	Baby Watercress	Crushed	5.56 ± 1.58	4.12 ± 0.44	1.85 ± 0.47
		Leek	Crushed	30.7 ± 8.40	24.4 ± 5.21	13.9 ± 3.52
		Aubergine	Crushed	39.6 ± 7.69	23.9 ± 2.77	17.8 ± 30.54
		Broccoli	Crushed	183 ± 13.8	84.9 ± 6.30	55.5 ± 1.71
		Onion	Crushed	30.0 ± 3.13	13.9 ± 1.75	9.90 ± 1.92
		Spinach	Crushed	1.22 ± 0.20	0.78 ± 0.13	0.54 ± 0.021
		Turnip	Crushed	9.45 ± 1.40	5.46 ± 0.18	—
	5 B	Peas	Food mill stainless steel	3.69 ± 3.86	1.87 ± 1.56	1.64 ± 0.75
		White beans		59.0 ± 9.43	44.0 ± 5.78	29.2 ± 2.64
		Chickpeas		51.7 ± 1.53	26.2 ± 1.36	17.3 ± 0.42
Level 6	6 A	Arborio Rice	Crushed	215.5 ± 7.22	—	—
		Carolino Rice	Crushed	76.1 ± 6.59	50.6 ± 4.30	37.8 ± 3.40
		Spaghetti	Crushed	—	—	—
	6 B	Chicken steak	Crushed	28.4 ± 2.88	11.8 ± 2.29	5.79 ± 0.29
		Meat Turkey steak	Crushed	35.9 ± 13.70	19.4 ± 2.88	10.9 ± 1.52
		Pork steak	Crushed	99.6 ± 19.73	70.3 ± 7.12	40.9 ± 5.78
		Beef steak	Crushed	9.60 ± 0.68	6.36 ± 0.51	3.42 ± 0.54

**Legend: 4 A** (easily crushable, no need to crush, no water added); **4 B** (easily crushable, no need to crush, water added); **4 C** (easily crushable, not homogeneous with some lumps, need to crush, no water added); **4 D** (easily crushable, not homogeneous with some lumps, need to crush, water added); **5 A** (kneaded with medium force (peel, flowers or leaves), not homogeneous, must be crushed, no water added); **5 B** (kneaded with force greater than 5A, skin present, must use cheesecloth); **6 A** (kneaded not homogeneous and forms a dense mass, must be crushed and water added); **6 B** (kneaded with great difficulty, leaving large pieces, must be crushed and water added).

**Table 5:** Comparison between the apparent viscosity of the cooked foods in tap water (TP) and the scale established based on the IDDSI values (*n* = 3).

IDDSI Levels		Food	Preparation	TP		
				*η* (Pa·s) 2.5 rpm	*η* (Pa·s) 5 rpm	*η* (Pa·s) 10 rpm
Level 4	4 A	Butternut squash	Mashed	88.8 ± 9.92	52.1 ± 15.44	30.9 ± 4.68
		Yellow squash	Mashed	18.2 ± 3.73	7.46 ± 3.62	5.44 ± 0.90
		Fuji apple	Mashed	37.7 ± 20.02	26.8 ± 6.47	13.8 ± 3.79
		Gala apple	Mashed	45.7 ± 6.27	19.0 ± 2.63	19.3 ± 0.72
		Golden apple	Mashed	55.9 ± 6.00	39.7 ± 3.76	20.9 ± 1.35
		Reineta apple	Mashed	78.7 ± 9.86	38.6 ± 7.27	19.8 ± 1.00
		Starking apple	Mashed	46.8 ± 5.31	35.8 ± 5.46	19.9 ± 2.11
		Rocha pear	Mashed	74.6 ± 8.42	46.3 ± 5.13	30.4 ± 3.62
	4 B	White potato	Mashed	97.4 ± 25.11	66.6 ± 8.42	36.7 ± 2.19
		Sweet potato	Mashed	55.4 ± 52.90	38.7 ± 29.1	23.9 ± 13.9
		Purple potato	Mashed	34.6 ± 9.04	25.4 ± 6.45	15.9 ± 3.86
		Turnip	Mashed	144.7 ± 60.3	100.8 ± 13.0	—
	4 C	Carrot	Crushed	86.8 ± 29.64	48.8 ± 6.88	31.7 ± 3.25
		Cauliflower	Crushed	4.75 ± 0.54	2.52 ± 0.40	1.43 ± 0.15
		Courgette	Crushed	11.1 ± 3.84	6.68 ± 1.69	4.05 ± 1.10
		Tomato	Crushed	30.2 ± 4.42	20.3 ± 1.95	—
	4 D	Catfish	Crushed	37.7 ± 3.69	19.8 ± 2.52	10.6 ± 1.60
		Tilapia fish	Crushed	24.6 ± 4.24	12.9 ± 2.03	6.42 ± 1.53
Level 5	5 A	Baby Watercress	Crushed	3.58 ± 0.35	2.08 ± 0.023	2.04 ± 0.32
		Leek	Crushed	23.8 ± 0.34	17.4 ± 5.23	12.8 ± 3.26
		Aubergine	Crushed	54.5 ± 0.68	36.6 ± 5.81	25.4 ± 2.67
		Broccoli	Crushed	210 ± 4.87	109 ± 8.88	—
		Onion	Crushed	24.2 ± 2.78	13.6 ± 0.21	7.32 ± 1.32
		Spinach	Crushed	1.19 ± 0.07	0.76 ± 0.16	0.41 ± 0.07
		Turnip	Crushed	8.80 ± 0.29	3.96 ± 0.92	2.34 ± 0.49
	5 B	Peas	Food mill stainless steel	7.39 ± 2.03	6.66 ± 0.05	4.64 ± 0.63
		White beans		68.9 ± 13.91	49.7 ± 3.38	34.9 ± 2.15
		Chickpeas		38.6 ± 10.94	45.1 ± 4.83	14.5 ± 10.11
Level 6	6 A	Arborio Rice	Crushed	221 ± 8.92	—	—
		Carolino Rice	Crushed	95.8 ± 11.27	67.8 ± 6.66	50.5 ± 3.81
		Spaghetti	Crushed	239.3	—	—
	6 B	Chicken steak	Crushed	0.57 ± 0.59	0.51 ± 0.18	0.18 ± 0.022
		Meat Turkey steak	Crushed	126.2 ± 61.33	41.9 ± 7.12	22.9 ± 2.12
		Pork steak	Crushed	58.2 ± 0.17	33.1 ± 3.31	12.4 ± 2.59
		Beef steak	Crushed	51.8 ± 27.83	79.1 ± 6.02	33.1 ± 6.45

**Legend: 4 A** (easily crushable, no need to crush, no water added); **4 B** (easily crushable, no need to crush, water added); **4 C** (easily crushable, not homogeneous with some lumps, need to crush, no water added); **4 D** (easily crushable, not homogeneous with some lumps, need to crush, water added); **5 A** (kneaded with medium force (peel, flowers or leaves), not homogeneous, must be crushed, no water added); **5 B** (kneaded with force greater than 5A, skin present, must use cheesecloth); **6 A** (kneaded not homogeneous and forms a dense mass, must be crushed and water added); **6 B** (kneaded with great difficulty, leaving large pieces, must be crushed and water added).

**Table 6:** Texture profile of each component of the proposed menus: soup (S), main course (M), and dessert (D).

Menu	Firmness (g)	Adhesiveness (g.s.)	Springiness	Cohesiveness	Gumminess (g)	Chewiness
S1	−0.426 ± 0.174	−2.625 ± 0.321	0.99 ± 0.0024	0.734 ± 0.009	−0.31 ± 0.127	−0.309 ± 0.126
S2	−0.257 ± 0.0502	−7.776 ± 0.234	0.99 ± 0.00323	0.641 ± 0.0202	−0.171 ± 0.0313	−0.169 ± 0.0312
S3	−0.399 ± 0.0753	−2.628 ± 0.841	0.99 ± 0.00514	0.755 ± 0.00914	−0.296 ± 0.0554	−0.294 ± 0.0544
S4	−0.304 ± 0.115	−4.665 ± 0.711	0.985 ± 0.00258	0.657 ± 0.0111	−0.205 ± 0.0752	−0.202 ± 0.0741
M1	−3.584 ± 0.192	−35.729 ± 0.515	0.976 ± 0.00243	0.663 ± 0.0353	−2.482 ± 0.100	−2.409 ± 0.0973
M2	−2.111 ± 0.0902	−16.337 ± 0.749	0.99 ± 0.00532	0.682 ± 0.0154	−1.439 ± 0.0963	−1.425 ± 0.0853
M3	−7.749 ± 0.331	−47.298 ± 7.835	0.913 ± 0.0183	0.848 ± 0.0632	−6.574 ± 0.233	−5.999 ± 0.327
M4	0.716 ± 0.091	−1.508 ± 0.688	0.869 ± 0.0691	0.547 ± 0.0164	0.392 ± 0.0384	0.312 ± 0.00415
D1	−2.249 ± 1.484	−20.52 ± 6.772	0.98 ± 0.00524	0.797 ± 0.0344	−1.793 ± 1.311	−1.758 ± 1.281
D2	0.682 ± 0.0681	−2.769 ± 1.577	0.757 ± 0.136	0.614 ± 0.0585	0.386 ± 0.0764	0.288 ± 0.115
D3	0.658 ± 0.216	−4.88 ± 4.954	0.985 ± 0.0691	0.723 ± 0.205	0.439 ± 0.205	0.426 ± 0.219
D4	−0.729 ± 0.166	−6.284 ± 1.094	0.985 ± 0.002	0.677 ± 0.0432	−0.478 ± 0.137	−0.471 ± 0.134

## Data Availability

The original contributions presented in this study are included in the article/Supplementary Material. Further inquiries can be directed to the corresponding author.

## Supplementary Materials

The following supporting information can be downloaded at https://www.mdpi.com/article/10.3390/foods15040708/s1, Table S1: Process of homogenizing cooked food; Table S2: The process of homogenising food consumed fresh (fruit); Table S3: Analytical methods used for water characterization; Table S4: Composition of the soups and their nutritional value. Soups were prepared in demineralized water (DW); Table S5: Composition of the main course and its nutritional value. Meals prepared in demineralized water (DW); Table S6: Composition of the dessert (fruit) and its nutritional value; Table S7: Characterization of demineralized water (DW) and tap water (TW); Table S8: pH and electric conductivity of demineralized water (DW) and tap water (TW) used to cook the foods; Table S9: Relationship between the rotational speed and shear rate.

## Abbreviations

The following abbreviations are used in this manuscript:

EC	Electrical Conductivity
DW	Desmineralized Water
TW	Tap Water
IDDSI	International Dysphagia Diet Standardisation Initiative
PAL	Physical-activity level
PDMS	Polydimethylsiloxane
TMD	Texture-modified diets
TPA	Texture Profile Analysis
