# Supplementary material for: Making Mealtime Easier: Nutrition and Texture in Foods for the Elderly with Swallowing Difficulties in Formal and Informal Care
Source: Foods. 2026 Feb 14;15(4):708. doi: 10.3390/foods15040708 (PMC12940075; doi:10.3390/foods15040708)
Supplement: Supplementary file 1 [file foods-15-00708-s001.zip › foods-4099319-supplementary.pdf]

Making Mealtime Easier: Nutrition and Texture in Foods for the Elderly with Swallowing Difficulties in Formal and Informal Care

Cristina M. M. Almeida [1], Juliana Beltrame [2], Joana Marto[1] and Lídia Pinheiro [1,*]

[1] Research Institute for Medicines (iMed.ULisboa), Faculty of Pharmacy, University of Lisbon, Portugal; calmeida@ff.ulisboa.pt (C.M.M.A.); jmmarto@ff.ulisboa.pt (J.M.)
[2] Faculty of Pharmacy, University of Lisbon, Portugal; julibnascimento@hotmail.com
* Correspondence: lpinheiro@ff.ulisboa.pt; Tel.: +351-217946400

Table S1. Process of homogenizing cooked food.

Processing	Food group	Food	Water (mL/ %)
Fork	Vegetables	butternut squash and yellow squash	---
		white potato	71/40
		sweet potato	53/40
		purple potato	44/40
		turnip	10/7
	Fruits	apples (Gala, Golden, Starking, Reineta, and Fugi) and Rocha pears	---
Electric food chopper*	Vegetables	baby watercress	---
		leek	---
		aubergine	---
		broccoli (flower)	---
		onion	---
		carrot	---
		courgette	---
		cauliflower	---
		spinach	---
		turnip greens	---
		tomatoes	---
	Cereals and derivatives, tubers	Carolino rice	47/25
		Arborio rice	47/25
		spaghetti	95/60
	Meat and Fish	chicken steak	71/71
		turkey steak	75/60
		pork steak	80/87
		beef steak	47/32
		catfish fillet	124/78
		Tilapia fillet	80/78
Manual food mill stainless steel	Pulses	peas	46/35
		white beans	25/15
		chickpeas	60/46

*Moulinex 1-2-3 (800 w)

Table S2. The process of homogenising food consumed fresh (fruit).

Processing	Food
Mashed (fork)	banana
Grated (Manual)	apples (Gala, Golden, Starking, Reineta and Fugi)
Crushed (glass mortar with pastel)	Rocha pear
Juice<br>Moulilex JU3701, Frutelia (Position 2)	apples (Gala, Golden, Starking, Reineta and Fugi)<br>Rocha pear<br>orange<br>clementine

Table S3. Analytical methods used for water characterization (Rice 2012).

Parameter (units)	Analytical method
pH	Potentiometric method, SMEWW 4500-H+
Turbidity (NTU)	Nephelometric method, SMEWW 2130 B
EC (μS/cm, 20 °C)	Potentiometric method, SMEWW 2510 B
Total hardness (mg/L $CaCO_3$)	Titrimetric method, SMEWW 2340 C

SMEWW – Standard method for the examination of water and wastewater

Table S4 Composition of the soups and their nutritional value. Soups were prepared in demineralized water (DW).

Soup (S)	Food group	Food	Weight (g)	g/100 g soup				Energy (kcal)/ 100 g)
				Carbohydrates (%)	Protein (%)	Lipids (%)	Fiber (%)	
Soup 1 (S1)	Tuber	White potato	125	4.58	0.59	---	0.40	21.5
	Vegetables	Baby watercress	70	0.53	0.26	0.08	0.10	4.1
		Onion	20	0.10	0.04	0.01	0.06	0.7
		Carrot	50	0.36	0.07	---	0.30	2.3
	Pulse	---	---					
	Fat	Olive oil	10	---	---	1.98	---	17.8
		DW	230	---	---	---	---	---
							**Total**	**46.4**
Soup 2 (S2)	Tuber	Red potatoes	125	3.35	0.39	0.01	0.21	15.5
	Vegetables	Leek	45	0.58	0.06	0.02	0.08	2.9
		Onion	20	0.08	0.03	0.01	0.05	0.6
		Cauliflowe r	60	0.23	0.16	0.02	0.18	2.1
		Courgette	45	0.21	0.09	0.03	0.08	1.6
		Turnip	55	0.21	0.04	0.04	0.21	1.8
	Pulse	---	---	---	---	---	---	---
	Fat	Olive oil	10	---	---	1.69	---	15.2
		DW	230	---	---	---	---	0.0
							**Total**	**39.6**
Soup 3 (S3)	Tuber	White potato	125	3.95	0.51	---	0.34	18.5
	Vegetables	Onion	20	0.08	0.03	0.01	0.05	0.6
		Carrot	50	0.31	0.06	---	0.26	2.0
		Turnip greens	70	0.52	0.14	0.03	0.42	3.7
	Pulse	White beans	80	2.00	0.90	0.07	0.92	14.0
	Fat	Olive oil	10	---	---	1.71	---	15.4
		DW	230	---	---	---	---	---
Soup 4 (S4)	Tuber	White potato	125	3.95	0.51	---	0.34	18.5
	Vegetables	Onion	20	0.08	0.03	0.01	0.05	0.6
		Yellow squash	60	0.41	0.19	0.06	0.25	3.5
		Courgette	60	0.28	0.12	0.04	0.10	2.1
	Pulse	Peas	80	1.03	0.77	0.07	1.00	9.8
	Fat	Olive oil	10	---	---	1.71	---	15.4
	DW	DW	230	---	---	---	---	---
							**Total**	**49.9**

*Values are calculated from the national food composition table; no experimental measurements or replicates were performed

Table S5. Composition of the main course and its nutritional value. Meals prepared in demineralized water (DW).

Main course	Food group	Food	Weight (g)	g/100 g				Energy* (kcal)/ 100 g)
				Carbohydrates (%)	Protein (%)	Lipids (%)	Fiber (%)	
Meal 1 (M1)	Tubers	---	---	---	---	---	---	---
	Vegetables	Butternut squash	80	5.34	0.58	0.10	2.01	28.6
		Carrot	60	2.16	0.42	0.00	1.80	13.9
		Broccoli	70	0.91	1.96	0.49	1.61	19.1
		Tomatoes	50	2.01	0.48	0.06	0.35	11.1
	Meat	Turkey	50	---	14.85	1.05	0.00	68.9
	Pulses	Chickpeas	80	13.36	6.72	1.68	4.08	103.6
	Fats	Olive oil	10	---	---	9.99	---	89.9
		Water	400	---	---	---	---	---
							**Total**	**335.1**
Meal 2 (M2)	Tubers	Red potatoes	125	19.75	2.31	0.05	1.25	91.2
	Vegetables	Onion	20	0.48	0.20	0.04	0.28	3.6
		Carrot	60	2.16	0.42	---	---	13.9
		Tomato	60	2.41	0.57	0.07	0.42	13.3
	Meat	Beef	50	---	10.55	1.75	---	58.0
	Pulses	Peas	80	6.00	4.48	0.40	5.84	57.2
	Fats	Olive oil	10	---	---	9.99	---	89.9
		Water	400	---	---	---	---	---
							**Total**	**327.2**
Meal 3 (M3)	Cereals	Curried rice	110	33.88	2.75	0.22	0.88	150.3
	Vegetables	Leek	40	3.05	0.32	0.08	0.40	15.0
		Onion	20	0.48	0.20	0.04	0.28	3.6
		Carrot	40	1.44	0.28	---	1.20	9.3
		Tomatoes	40	1.60	0.38	0.04	0.28	8.9
	Fish	Tilapia fish	50	---	11.10	1.04	---	53.7
	Pulses	---	---	---	---	---	---	---
	Fats	Olive Oil	10	---	---	9.99	---	89.9
		Water	400	---	---	---	---	---
							**Total**	**330.7**
Meal 4 (M4)	Tubers	Sweet potatoes	125	35.38	1.25	---	3.75	154.0
	Vegetables	Onion	20	0.48	0.20	0.04	0.28	3.6
		Cauliflower	45	1.04	0.72	0.09	0.81	9.5
	Fish	Catfish	50	---	8.75	0.50	---	39.5
	Pulses	---	---	---	---	---	---	---
	Fats	Olive oil	10	---	---	9.99	---	89.9
		Water	400	---	---	---	---	---
							**Total**	**296.5**

*Values are calculated from the national food composition table; no experimental measurements or replicates were performed

Table S6 Composition of the dessert (fruit) and its nutritional value.

Dessert	Food group	Food	Weight (g)	g/100 g				Energy* (kcal)/ 100 g)
				Carbohydrates (%)	Protein (%)	Lipids (%)	Fiber (%)	
Dessert (D1)	Fruit	Cooked Gala apple	170	23.29	0.43	0.20	3.91	104.5
Dessert (D2)	Fruit	Cooked Rocha pear	140	13.16	0.42	0.56	2.52	64.4
Dessert (D3)	Fruit	Cooked Golden apple	170	23.12	0.48	0.26	4.08	104.8
Dessert (D4)	Fruit	Ripe banana	170	37.06	2.72	0.68	5.27	175.8

*Values are calculated from the national food composition table; no experimental measurements or replicates were performed

Table S7. Characterization of demineralized water (DW) and tap water (TW).

Parameters	DW	TW
pH@20 °C	5.88 ± 0.18	7.83±0.18
EC, μS/cm@20 °C	0.64 ± 0.18	150 ± 0.19
Turbidity, NTU	0.12 ± 0.04	0.25 ± 0.07
Total hardness, mg/L $CaCO_3$	20 ± 2.3	80 ± 8.3
Viscosity@25 °C, 30 rpm, Pa·s	$1.1\times10^{-3} \pm 0.07\text{x}10^{-3}$	$1.2 \times10^{-3} \pm 0.11\text{x}10^{-3}$

Table S8. pH and electric conductivity of demineralized water (DW) and tap water (TW) used to cook the foods.

Cooked Food	DW		TW	
	pH@20 °C	EC, mS/cm@20 °C	pH@20 °C	EC, mS/cm@20 °C
Arborio rice	5.5 ± 0.010	0.35 ± 0.010	6.1 ± 0.0050	0.46 ± 0.0020
Aubergine	5.1 ± 0.010	1.6 ± 0.050	5.6 ± 0.030	1.5 ± 0.040
Baby watercress	6.2 ± 0.0011	3.4 ± 0.010	6.10 ± 0.0010	3.5 ± 0.010
Broccoli (flower)	6.0 ± 0.010	2.4 ± 0.010	6.3 ± 0.0060	2.6 ± 0.0090
Carolino Rice	5.9 ± 0.18	0.64 ± 0.18	7.8 ± 0.18	0.85 ± 0.18
Carrot	5.5 ± 0.0012	5.7 ± 0.0020	5.6 ± 0.0054	7.2 ± 0.0010
Catfish fillets	8.6 ± 0.080	5.5 ± 0.044	8.7 ± 0.072	5.1 ± 0.010
Cauliflower (flower)	6.5 ± 0.012	2.1 ± 0.012	6.9 ± 0.0063	2.1 ± 0.010
Chicken steak	6.0 ± 0.020	5.4 ± 0.012	6.1 ± 0.011	5.6 ± 0.030
Chickpeas	6.0 ± 0.010	4.5 ± 0.023	6.1 ± 0.0010	5.5 ± 0.010
Courgette	5.9 ± 0.0010	1.9 ± 0.014	6.2 ± 0.0050	1.9 ± 0.0050
Fugi apple	3.6 ± 0.0060	0.61 ± 0.0010	3.7 ± 0.050	0.74 ± 0.0030
Gala apple	4.1 ± 0.012	0.91 ± 0.015	4.1 ± 0.010	0.84 ± 0.0020
Golden apple	3.6 ± 0.0061	0.86 ± 0.0052	3.7 ± 0.0060	1.1 ± 0.0060
Leek	5.8 ± 0.010	1.5 ± 0.043	6.4 ± 0.0060	1.8 ± 0.0010
Minced rump steak	6.3 ± 0.0011	4.1 ± 0.052	6.2 ± 0.0010	4.8± 0.020
Onion	5.4 ± 0.010	2.2 ± 0.0050	5.4 ± 0.010	1.7 ± 0.0040
Peas	6.6 ± 0.0060	3.1 ± 0.0050	6.6 ± 0.012	4.3 ± 0.12
Pork steak	5.9 ± 0.0051	6.1 ± 0.060	6.1 ± 0.0060	7.0± 0.0
Purple potato	6.0 ± 0.011	3.4 ± 0.0051	6.8 ± 0.43	3.6 ± 0.0050
Reineta apple	3.4 ± 0.010	0.98 ± 0.0043	3.5 ± 0.0060	0.83 ± 0.0010
Rocha pear	4.6 ± 0.010	0.77 ± 0.0042	4.8 ± 0.0060	1.4 ± 0.0040
Spaghetti	5.7 ± 0.0060	2.1 ± 0.042	5.9 ± 0.040	2.2± 0.070
Spinach	6.6 ± 0.0010	5.7 ± 0.031	6.52 ± 0.010	6.7 ± 0.0050
Squash (Yellow)	5.8 ± 0.19	3.6 ± 0.010	6.2± 0.020	3.7 ± 0.040
Squash (Butternut)	5.6 ± 0.0012	2.1 ± 0.011	5.9± 0.012	2.2 ± 0.010
Starking apple	4.1 ± 0.0062	0.49 ± 0.0024	4.3 ± 0.0010	0.56 ± 0.0020
Sweet potato	5.8 ± 0.013	1.8 ± 0.0010	6.3 ± 0.0060	1.9 ± 0.0090
Tilapia fillets	6.9 ± 0.0061	4.5 ± 0.022	7.10± 0.020	4.3± 0.051
Tomato	4.5 ± 0.010	1.5 ± 0.0053	4.63 ± 0.01	1.5± 0.0032
Turkey steak	6.10 ± 0.005	5.5 ± 0.052	6.05 ± 0.02	5.7 ± 0.0010
Turnip	5.9 ± 0.0060	2.3 ± 0.010	6.58 ± 0.02	2.2 ± 0.010
Turnip greens	5.8 ± 0.010	3.1 ± 0.020	5.96 ± 0.00	3.5 ± 0.0010
White beans	6.0 ± 0.0060	4.7 ± 0.040	6.13 ± 0.01	4.7 ± 0.033
White potato	5.6 ± 0.030	5.6 ± 0.010	5.84 ± 0.03	6.6 ± 0.022

Table S9. Relationship between the rotational speed and shear rate.

Rotational speed (rpm)	Shear rate (s^{-1})
2.5	0.5
5	1
10	2
50	10
100	20
